# Seronegative MSM at high risk of HIV-1 acquisition show an immune quiescent profile with a normal immune response against common antigens

**DOI:** 10.1371/journal.pone.0277120

**Published:** 2022-12-08

**Authors:** Ana C. Ossa-Giraldo, Yurany Blanquiceth, Lizdany Flórez-Álvarez, Katherin Contreras-Ramírez, Mauricio Rojas, Juan C. Hernandez, Wildeman Zapata

**Affiliations:** 1 Grupo Infettare, Facultad de Medicina, Universidad Cooperativa de Colombia, Medellín, Antioquia, Colombia; 2 Grupo Inmunovirología, Facultad de Medicina, Universidad de Antioquia UdeA, Medellín, Antioquia, Colombia; 3 Department of Parasitology, Institute of Biomedical Sciences at the University of São Paulo, São Paulo, Brazil; 4 Grupo de Inmunología Celular e Inmunogenética, Sede de Investigación Universitaria (SIU), Universidad de Antioquia, UdeA, Medellín, Antioquia, Colombia; 5 Unidad de Citometría de Flujo, Sede de Investigación Universitaria (SIU), Universidad de Antioquia UdeA, Medellín, Antioquia, Colombia; Public Health Agency of Canada, CANADA

## Abstract

Human immunodeficiency virus (HIV) infection still represents a major public health problem worldwide, and its vaccine remains elusive. The study of HIV-exposed seronegative individuals (HESN) brings important information about the natural resistance to HIV, allows a better understanding of the infection, and opens doors for new preventive and therapeutic strategies. Among HESN groups, there are some men who have sex with men (MSM) with high-risk sexual behaviors, who represent an adequate cohort for HESN study because of their major HIV exposure without infection. This study aimed to compare the immunological profile of Colombian seronegative MSM with different risk sexual behaviors. This study included 60 MSM at high-risk (n = 16) and low-risk (n = 44) of HIV-1 acquisition. No sex worker nor homozygous delta 32 mutation subjects were included. All participants were negative for anti-HIV-1/2 antibodies and HIV-1 proviral DNA. A higher frequency of sexual partners in the last 3 months before the study participation (median, 30 vs. 2), lifetime sexual partners (median, 1,708 vs. 26), and unprotected anal intercourse (median 12.5 vs. 2) was determined in high-risk MSM than low-risk MSM. High-risk MSM also showed a quiescent profile of T cells and natural killer (NK) cells, with a significantly lower percentage of CD4+CD38+, CD4+HLADR−CD38+, CD4+Ki67+ T cells, and NKG2D+ NK cells (CD3−CD16+CD56+), a significantly higher percentage of CD4+HLADR−CD38−, and a tendency to show a higher percentage of CD8+HLADR+CD38− T cells than the low-risk group. Likewise, they showed higher mRNA levels of Serpin A1 from PBMCs. The results suggest that this MSM cohort could be HESN individuals and their resistance would be explained by a quiescent profile of T cells and NK cells and an increased Serpin A1 expression. Further study on MSM at high risk of exposure to HIV-1 is necessary to better understand the natural resistance to HIV.

## Introduction

HIV-1 infection remains to be a big health issue and priority worldwide, despite the availability of highly effective antiretroviral therapy [1]. Several efforts have been made to find a vaccine but it continues elusive [2]. Some people (HIV-exposed seronegative [HESN]) exhibit a natural resistance to HIV-1, and persist without an established infection besides their viral exposure [3]. The comprehension of HESN’s biological phenomena is an advantage to better understand the HIV-1 infection and brings new options for therapeutic and preventive strategy designs [3, 4]. Since the first reports of natural resistance to HIV-1 [5, 6], the mechanisms that can explain this phenomenon in HESN individuals are a major concern for HIV researchers, and still current now.

The main factors associated with HIV natural resistance include the homozygous deletion of 32bp in CCR5 co-receptor or Δ32 mutation, which is present in nearly 1% of the global population [7], and recently described, the heterozygous single nucleotide deletion in the stop codon of the nuclear import factor Transportin 3 gene (*TNPO3*) [8]. However, HESN individuals have been observed who do not have the homozygous Δ32 mutation and show a relative natural resistance to HIV-1. Immunological and genetic factors such as the overexpression of antimicrobial peptides in the mucosa, increased activity of NK cells, a combination of certain HLA-KIR genotypes, and HIV-1-specific CTL (cytotoxic T cells) responses have been associated with the possible HIV natural resistance in those individuals [9–13].

In the context of HIV-1 exposure, the presence of activated cells and inflammation at the site of HIV entry increase the risk of infection as reported in people with clinical or subclinical sexually transmitted infections (STIs) and mucosal inflammatory process [14–17]. The immune quiescence, characterized by a low target cell activation profile has been proposed as a protection mechanism against HIV infection and is found in several HESN cohorts, including men who have sex with men (MSM) [18–22]. Previous studies in the Pumwani cohort of well-studied HESN African female sex workers revealed a relationship between the reduced susceptibility to HIV-1 infection to a lower frequency of activated CD4+CD69+ T cells and an elevated number of regulatory T (Treg) cells [23]. The low expression of genes implicated in T cells receptor signaling and HIV host-dependent factors also have been described together with lower levels of secreted cytokines at basal state, but not after stimulation, which shows an important difference between quiescence and immunosuppression [24, 25]. Contrary to this, other studies have shown that immune activation is related to protection. Biasin *et al*. (2000) reported an increased expression of proinflammatory cytokines and chemokine receptors in cervical biopsies of HESN women [26]. Increased memory and activated T cells have also been described [27, 28]. These results revealed the unclear role of cell activation in HIV-1 protection.

From the first reports of resistance in 1989 [6] to the present, MSM has represented an adequate cohort for the HESN study. The high prevalence of HIV in this population [1] and the higher probability to acquire the infection through anal sex [29] put MSM, who practice risky sexual behaviors, such as anal sex without a condom and having multiple partners, at extreme risk of HIV exposure. Therefore, the study of seronegative MSM with high-risk behaviors and the possible findings of HESN individuals in this population represents an important opportunity to better understand natural resistance to HIV.

The HIV prevalence in Colombian MSM is ~43 times higher than in the general population [17% vs. 0.4%] [1], and pre-exposure prophylaxis (PrEP) has not yet been approved in the country. Therefore, Colombian seronegative MSM that practice high-risk sexual behaviors are a very interesting population to address the mechanisms underlying HIV natural resistance. However, engaging this population in Latin America is not easy because most MSM does not want to share private information or even be identified due to the historic oppression they have suffered. This study aimed to compare the immunological profile of Colombian HIV seronegative MSM.

## Material and methods

### Study population

This study included 60 participants from an MSM cohort from Medellín-Colombia who signed the informed consent and accepted to participate in the study. Participants were recruited using a combination of several methods to sample hard-to-reach populations, and sociodemographic data and sexual behaviors were defined by structural surveys and in-depth interviews, as previously reported [30]. Participants were classified into two groups according to the frequency of sexual partners in the last 3 months before the study participation. The high-risk group (n = 16) was defined as MSM with >14 sexual partners and the low-risk group (n = 44) were MSM with four or fewer sexual partners in the last 3 months. All individuals met the following inclusion/exclusion criteria: no one was a sex worker nor taking PrEP and all were negative for anti-HIV-1 antibodies, HIV-1 proviral DNA, and delta 32 mutation in the *CCR5* gene in a homozygous state. The study was performed according to the Helsinki declaration and was approved by the Ethics Committee of Universidad de Antioquia’s School of Medicine (Act No.007, May 22th, 2014).

### Biological samples

Peripheral blood and anal mucosa samples were obtained from each subject; the whole blood was used to obtain plasma and serum, to extract DNA, as well as for peripheral blood mononuclear cell (PBMC) isolation. The anal mucosa sample was used for cytology analysis and mRNA extraction and is collected using an optimized protocol as follows: the cytobrush was inserted 5 cm beyond the anal verge, close to the anal wall, and rotated slowly while being withdrawn to capture cells. Then, the sample was spread onto slides for cytology analysis and the cytobrush’s head was cut and put into a vial with RNAlater reagent. A second cytobrush was inserted to obtain more mucosal samples, and this head was put into an RNAlater reagent (Invitrogen). Samples were preserved at 4°C. Then, the cytobrush’s heads were removed and the cell pellet was obtained by centrifugation. TRIzol Reagent (Zymo) was added to lyse the cells. The samples were preserved at −80°C until RNA extraction. The anal sampling and cytology analysis was done by qualified staff from the reference lab “Laboratorio Clínico VID.”

### T cell basal activation profile

Fresh whole blood was stained for 25 min at room temperature in the dark with the monoclonal antibodies anti-CD4-PerCp-Cy5.5 clone OKT4, anti-CD8-eFluor 450 clone OKT8, anti-HLA-DR-FITC clone LN3, anti-CD69-APC clone FN50, anti-CD38-PE-Cy7 clone HIT2, and fixable viability dye-eFluor 506 (Thermo Fisher Scientific, Wilmington, DE, United States). Erythrocytes were lysed (BD FACS Lysing Solution, BD Biosciences, San Jose, CA, United States) following the manufacturer’s instructions, and then the cells were permeabilizated and stained with anti- Ki-67-PE clone B56 (BD Biosciences, San Jose, CA, United States) and anti-CD3-Alexa eFluor 700 clone UCHT1 (Thermo Fisher Scientific, Wilmington, DE, United States) for 25 min at 4°C in the dark. The cells were fixed with 1% formaldehyde, acquired using a BD LSRFortessa™ flow cytometer, and, analyzed in FlowJo software (Becton–Dickinson, San Diego, CA, USA).

### HIV-1-specific T cell responses

PBMCs were isolated using density-gradient with Ficoll-Hypaque (Sigma-Aldrich, St. Louis, MO, United States), washed with phosphate-buffered saline (Lonza, Rockland, ME, United States), and suspended in RPMI medium (Lonza, Rockland, ME, United States) supplemented with 10% fetal bovine serum (Gibco, Grand Island, NY, United States) and 1% penicillin/streptomycin (Thermo Fisher Scientific, Wilmington, DE, United States). The cells were cultured in the presence of 3 μg/mL of brefeldin A, 2 uM of monensin, 1ug/mL of anti-CD28 clone CD28.2, and 1ug/mL of anti-CD49d clone 9F10 (Thermo Fisher Scientific, Wilmington, DE, United States), and stimulated overnight with a pool of peptides from HIV-1 subtype B consensus Gag at a final concentration of 5μg/mL (National Institutes of Health, AIDS Research, and Reference Reagents Program). *Staphylococcus* enterotoxin B (SEB) was used as the positive control. After the overnight stimulus, the supernatants were collected and stored at −80°C until the cytometric bead array (CBA) assay. The cells were stained with monoclonal antibodies CD3-Alexa eFluor 700 clone UCHT1, CD4-eFluor 660 clone OKT4, CD8-eFluor 450 clone OKT8, Tumor necrosis factor α (TNF-α)-PerCp-Cy5.5 clone Mab11, fixable viability dye-eFluor 506 (Thermo Fisher Scientific, Wilmington, DE, United States), interferon-gamma (IFN-γ)-brilliant Violet 711 clone 4S.B3 (BioLegend INC, San Diego, CA), Granzyme B-FITC clone GB11, and MIP1-β-PE clone D21-1351 (BD Biosciences, San Jose, CA, United States) for 25 min at 4°C in the dark. After staining with extracellular markers, the cells were permeabilized and fixed using the Intracellular Fixation & Permeabilization Buffer Set (eBioscience™) following the manufacturer’s instructions. The cells were then incubated for 25 minutes at 4°C, washed twice with 2 mL of phosphate-buffered saline at 250g for 5 minutes, and fixed with 1% formaldehyde. Data were acquired with a BD LSRFortessa™ flow cytometer and analyzed in FlowJo software (Becton–Dickinson, San Diego, CA, USA). In the cytometry analysis, cytokine expression data were reported after the background subtraction. The Boolean gate analysis for polyfunctional evaluation was performed with the SPICE software (version 6.0; NIH, Bethesda, MD). The level of 0.1% was considered as the threshold for a positive response for all cytokines. The INDEX of polyfunctionality (pINDEX) was calculated with Funky Cells software (Boyd, et al. [31]).

### NK cell profile

PBMCs were stimulated with interleukin (IL)-12 and IL-15 (20 μg/mL) for 48 h, and 3 μg/mL brefeldin A and 2 uM monensin were added 24 h post culture. Cells were stained with monoclonal antibodies CD16-Alexa Fluor 647 clone 38G (BioLegend INC, San Diego, CA), CD56- PE-Cy5 clone CMSSB, NKG2D-PerCP-eFluor710 clone 1D11, and fixable viability dye-eFluor 506 (Thermo Fisher Scientific, Wilmington, DE, United States) for 25 min at room temperature in the dark. Then, the cells were permeabilized and stained with CD3-Alexa eFluor 700 clone UCHT1 (Thermo Fisher Scientific, Wilmington, DE, United States), IFN-γ -Brilliant Violet 711 clone 4SB3 (BioLegend INC, San Diego, CA), Granzyme-FITC clone GB11 and, Perforin-PE (BD Biosciences, San Jose, CA, United States), for 25 min at 4°C in the dark. After staining with extracellular markers, the cells were permeabilized and fixed using the Intracellular Fixation & Permeabilization Buffer Set (eBioscience™) following the manufacturer’s instructions. The cells were then incubated for 25 minutes at 4°C, washed twice with 2 mL of phosphate-buffered saline at 250g for 5 minutes and fixed with 1% formaldehyde. Data were acquired with a BD LSRFortessa™ flow cytometer and analyzed in FlowJo software (Becton–Dickinson, San Diego, CA, USA).

### mRNA quantification by real-time reverse transcription-polymerase chain reaction (RT-PCR) from PBMC and mucosal samples

Total RNA was purified using the Direct-zol RNA Miniprep kit (Zymo Research), treated with DNase, and retrotranscribed to cDNA using the High-Capacity cDNA Reverse Transcription Kit (Thermo Fisher Scientific, Wilmington, DE, United States). PCR reactions were performed using the Maxima SYBR Green qPCR master mix kit (Fermentas). The specific primers and PCR conditions are shown in Tables 1 and 2 in the S1 Text. Real-time RT-PCR was performed in a QuantStudio 5 Real-Time PCR System (Thermo Fisher Scientific, Wilmington, DE, United States). The data are expressed as mRNA relative units of each gene that are normalized against the constitutive gene phosphoglycerate kinase (PGK) using the formula: 1.8^−[ΔCt]^, where 1.8 corresponds to the mean PCR efficiency of 90%.

### Cytokines quantification by CBA

The IL-1β, IL-6, IL-8, IL-12p70, TNF-α, and IL-10 levels were analyzed in plasma and CTL culture supernatants. Cytokine concentration was determined by BD™ CBA Human Inflammation Kit (Becton–Dickinson, San Diego, CA, USA), following the manufacturer’s instructions. The data were analyzed in BD LSR Fortessa™ flow cytometer and FlowJo software (Becton–Dickinson, San Diego, CA, USA).

## Results

### The MSM from the high-risk group show risky sexual behaviors and remain HIV seronegative

The median age was 24.5 and 32 years for the low and high-risk groups, respectively. In both groups, most participants identified themselves as gay/homosexual males, were single, and had access to vocational/professional education. The MSM in the high-risk group showed riskier sexual behaviors, including a higher number of sexual partners in the last 3 months (*p* < 0.001), a higher number of lifetime sexual partners (*p* < 0.001), more unprotected intercourses in the last 3 months (*p* = 0.001), and lower frequency of protected intercourses with their regular partners (*p* = 0.001) compared with the low-risk group. Additionally, in the high-risk group, 81.3% practice receptive or versatile roles, 81.3% never use condoms with their casual partners, 87.5% reported a history of STI, and 100% seek casual sex at bathhouses and clubs. The sociodemographic and sexual behavior data of MSM groups are described in Table 1.

**Table 1:** Sociodemographic and sexual behavior data of the low- and high-risk groups of MSM.

Variable	Low-risk^a^	High-risk	*p*-value
	(n = 44)	(n = 16)	
**Sociodemographic data**			
Age			**0.002** ^a^
Median (IQR)	24.5 (20–29)	32 (26.5–36.7)	
	**Low-risk**	**High-risk**	***p*-value**
	**n (%)**	**n (%)**	
Gender identity			NA
Male	43 (97.7)	16 (100)	
Queer	1 (2.3)	-	
Educational level			**<0.001** ^c^
High School	3 (7.0)	1 (7)	
Vocational/Undergraduate education	41 (93)	15 (94)	
Occupation			**0.026** ^c^
Student	15 (34.1)	-	
Student and employed	5 (11.4)	-	
Employed Professional	17 (38.6)	11 (68.8)	
Unemployed Professional	1 (2.3)	1 (6.3)	
Employed/other not professional services^d^	6 (13.6)	4 (25)	
Marital status			NA
Single	42 (95.5)	14 (87.5)	
Cohabiting	2 (4.5)	2 (12.5)	
**Sexual Behavior**			
Sexual orientation			NA
Bisexual	4 (9.1)	3 (18.8)	
Gay/Homosexual	38 (86.4)	13 (81.3)	
Pansexual	1 (2.3)	-	
Age of sexual debut			0.36^c^
6–9 years old	4 (9.2)	1 (6.3)	
10–14 years old	8 (18.2)	5 (31.3)	
15–17 years old	18 (40.9)	5 (31.3)	
18 and older	14 (31.8)	5 (31.3)	
Type of sexual partners			NA
Males and Women	-	1 (6.3)	
Only Men	44 (100)	15 (93.8)	
Sexual role			NA
Insertive	3 (6.8)	3 (18.8)	
Receptive	10 (20.5)	1 (6.3)	
Versatile	31 (70.5)	12 (75)	
Protected sexual intercourse with regular partners			**0.001** ^c^
Always	9 (20.5)	-	
Never	33 (75)	15 (93.8)	
Protected sexual intercourse with casual partners			0.29^c^
Always	22 (50)	3 (18.8)	
Never	20 (45.5)	13 (81.3)	
Casual sex (bathhouses, clubs, seeking sex by social media)			NA
Yes	9 (20.5)	16 (100)	
No	35 (79.5)	-	
Last unprotected sexual intercourse			NA
Never	6 (13.6)	1 (6.2)	
<1 month	12 (27.3)	7 (43.7)	
1–2 months	9 (20.5)	3 (18.8)	
3–5 months	6 (13.6)	3 (18.8)	
6–11 months	4 (9.1)	-	
≥1 year	7 (15.9)	2 (12.5)	
Reported previous STIs	14 (31.8)	14 (87.5)	0.6^c^
Type of reported STIs			NA
Hepatitis B	-	2 (14.3)	
Gonorrhea	5 (35.7)	7 (50)	
Syphilis	3 (21.4)	6 (42.9)	
Herpes	1 (7.1)	1 (7.1)	
PVH	7 (50)	2 (14.3)	
Not specified	-	2 (14.3)	
Frequency of HIV positive sexual partners	16 (36.6)	9 (56.2)	0.16^c^
Number of sexual partners in the last 3 months			**<0.001** ^a^
Median (IQR)	2 (1–4)	31 (23–45)	
Number of unprotected sexual intercourses in the last 3 months			**0.001** ^a^
Median (IQR)	2 (0–2)	10 (6–24)	
Number of lifetime sexual partners			**<0.001** ^a^
Median (IQR)	26 (11–97)	1078 (900–4100)	

^a^Subjects in the low-risk group exhibit a lower risk of HIV infection in comparison with those in the high-risk group.

^b^Mann–Whitney U-test.

^c^Chi-squared test.

^d^ Refers to jobs that do not require professional qualifications.

NA: not applicable, not analyzed

### Seronegative MSM at high risk of HIV-1 infection show a low T cell activation profile and a higher expression of Serpin A1

**Fig 1:**
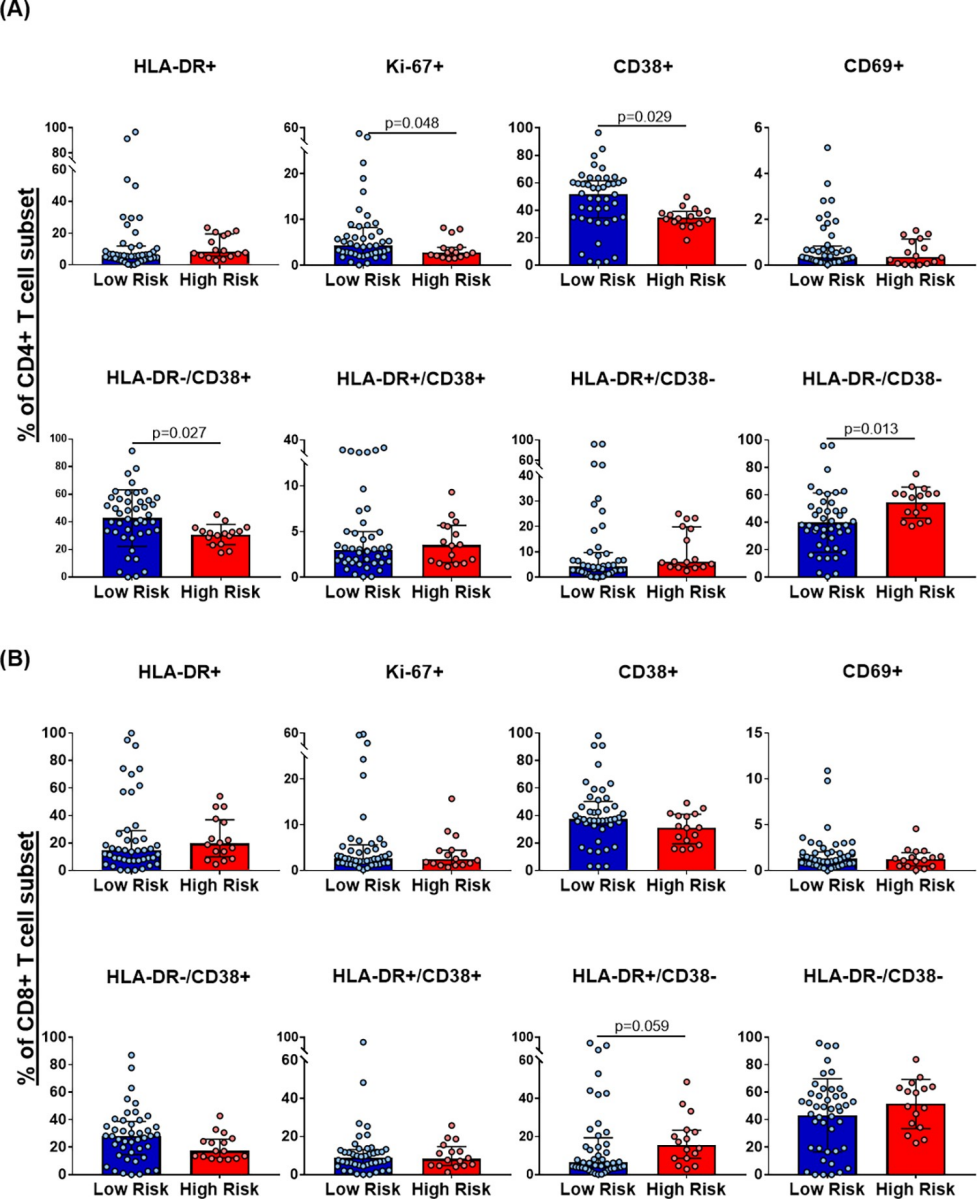
CD4+ and CD8+ T cells basal activation profile in both MSM groups. Comparison of the percentage of **(A)** CD4+ and **(B)** CD8+ T cells expressing HLA-DR, Ki-67, CD38, and CD69 activation markers between the groups. High-risk group (n = 16), low-risk group (n = 44). All data are reported after background correction. The comparison was performed with the Mann–Whitney U-test or Student T-test according to the data distribution.

The percentage of cells expressing CD38, HLA-DR, CD69, and Ki-67 molecules was quantified by flow cytometry to explore the basal activation profile of CD4+ and CD8+ T cells. The high-risk group showed a low activation profile in T cells with lower percentage of CD4+CD38+ (*p* = 0.002), CD4+HLADR-CD38+ (*p* = 0.027), and CD4+Ki67+ cells (*p* = 0.048). Likewise, this group had a higher percentage of CD4+HLADR−CD38− (*p* = 0.013). The high-risk group seemed to present a lower percentage of CD8+HLADR+CD38− cells although it was not statistically significant (*p* = 0.058). No differences were found in CD69 expression between both groups (Fig 1). The concentrations of IL-6, IL-8 IL-10, IL-12p70, and TNF-α were quantified by CBA, and the mRNA expression of Elafin, Serpin A1, MIP1-β, RANTES, IL-1β, IL-18, IL-22, Caspase 1, and FoxP3, in PBMCs through qPCR to identify the differences in the basal levels of plasmatic cytokines and soluble factors between both MSM groups. The MSM in the high-risk group exhibited higher mRNA levels of Serpin A1 (*p* = 0.018) and a tendency to express more MIP1-β (0.27 relative mRNA units vs. 0.10 relative mRNA units; *p* = 0.06) compared with the low-risk group. No differences were found in the other evaluated soluble factors (Tables 2 and 3).

**Table 2:** Plasma levels of cytokines in both groups of MSM.

Cytokine	Low-risk (n = 44)	High-risk (n = 16)	Mann–Whitney U-test
	pg/mL Median (IQR)	pg/mL Median (IQR)	*p*-value
**IL-6**	13.9 (12.8–15.0)	14.0 (12.9–14.9)	0.95
**IL-8**	24.3 (23.0–26.2)	26.1 (24.4–29.1)	0.08
**IL-10**	15.7 (15.4–15.9)	15.5 (15.4–15.6)	0.23
**IL-12**	17.4 (17.0–19.0)	17.1 (16.8–17.5)	0.10
**TNF**-**α**	0.0 (0.0–4.7)	0.0 (0.0–3.9)	0.61

**Table 3:** mRNA gene expression in PBMCs of both MSM groups.

Gene	Low-Risk	High-Risk	Mann–Whitney U-test
	(n = 29)	(n = 9)	
	mRNA relative units	RFU Median (IQR)	*p*-value
	Median (IQR)		
**Caspase 1**	0.285 (0.0634–0.174)	0.330 (0.174–0.534)	0.49
**Elafin**	0.0003 (0.0001–0.0006)	0.0005 (0.0001–0.0012)	0.51
**IL-1β**	0.334 (0.167–0.855)	0.334 (0.167–0.855)	0.79
**IL-18**	0.004 (0.002–0.007)	0.004 (0.003–0.008)	0.82
**Serpin A1**	1.612 (0.785–2.750)	2.326 (1.390–3.456)	**0.02**
**FoxP3**	0.005 (0.001–0.012)	0.005 (0.001–0.011)	0.76
**IL-22**	0.0005 (0.0003–0.0012)	0.0004 (0.0003–0.0006)	0.74
**MIP1-β**	0.130 (0.059–0.237)	0.245 (0.102–0.349)	0.06
**RANTES**	1.223 (0.473–1.839)	1.258 (0.604–2.370)	0.15

Data are expressed as mRNA relative units of each gene (median and interquartile range [IQR]) normalized against the constitutive gene PGK.

### No differences were found in the T cell response to HIV-1 Gag peptides between both MSM groups

**Fig 2:**
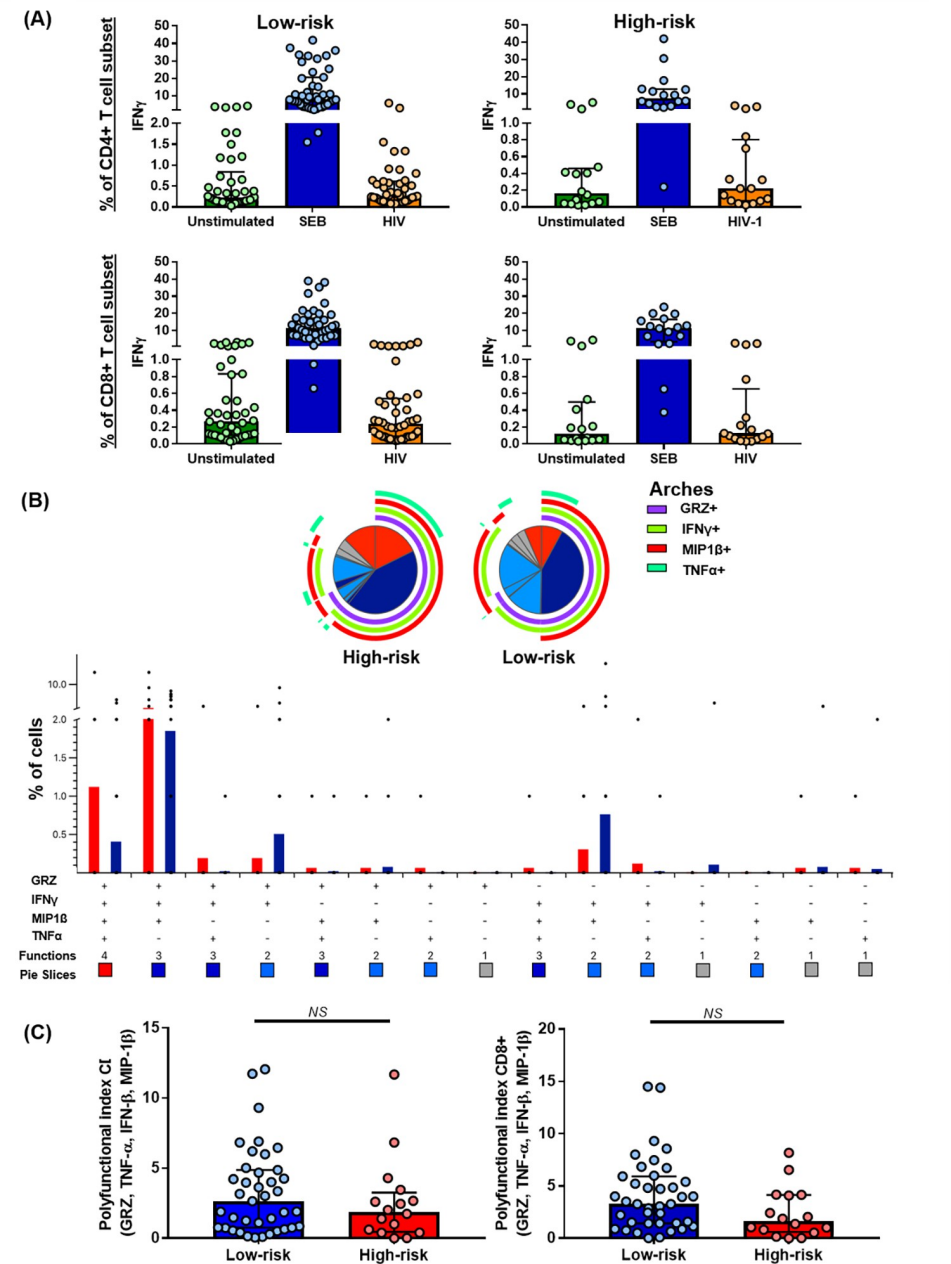
T cell responses against a pool of HIV-1 Gag peptides. **(A)** IFN-γ expression by unstimulated CD4+ and CD8+ T cells, or after SEB and HIV-1 Gag peptides stimulus. **(B)** Comparison of the polyfunctional profiles of Gag-specific responses in CD4+ T cells from both MSM groups. The slices of the pies correspond to the proportions of Gag-specific CD4+ T cells expressing 1 (grey), 2 (light blue), 3 (dark blue), or 4 (red) functions, until n simultaneous parameters (n + 1 dimensions) are calculated using the Boolean gating; the results are presented as mean. In the permutation analysis conducted in the SPICE platform, only data higher than 0.1% were included (after background subtraction). **(C)** INDEX of polyfunctionality (pINDEX) of CD4+ and CD8+ T cells from both MSM groups based on the proportions of cells producing intracellular combinations of Granzyme B, MIP1-β, TNF-α, and IFN-γ; the pINDEX was calculated with Funky Cells software [31]; the comparison was performed with the Mann–Whitney U-test. For all the panels: high-risk group n = 16, low-risk group n = 44.

The intracellular expression of Granzyme B, MIP1-β, TNF-α, and IFN-γ by CD4+ and CD8+ T cells was measured after stimulus with a pool of peptides from the HIV-1 subtype B consensus Gag or SEB (Fig 2 in S1 Text). Likewise, the IL-10, IL-12, IL-1b, IL-6, IL-8, and TNF-α levels in the culture’s supernatants were quantified by CBA. All the 60 MSM showed a strong response to the superantigen SEB (used as a positive control for T-cell activation); however, the HIV-1 specific response was detected in some participants and a defined pattern of response by risk group was not found. No differences were found in the magnitude nor index of T cell polyfunctionality between the MSM groups in response to HIV-1 Gag peptides (Fig 2). Finally, no statistical differences were found between the groups in the IL-10, IL-12, IL-1β IL-6, IL-8, and TNF-α levels in the supernatants of HIV-1-stimulated cells (Table 3 in S1 Text).

### Seronegative MSM at high risk of HIV-1 infection show a low expression of NKG2D on NK cells

**Fig 3:**
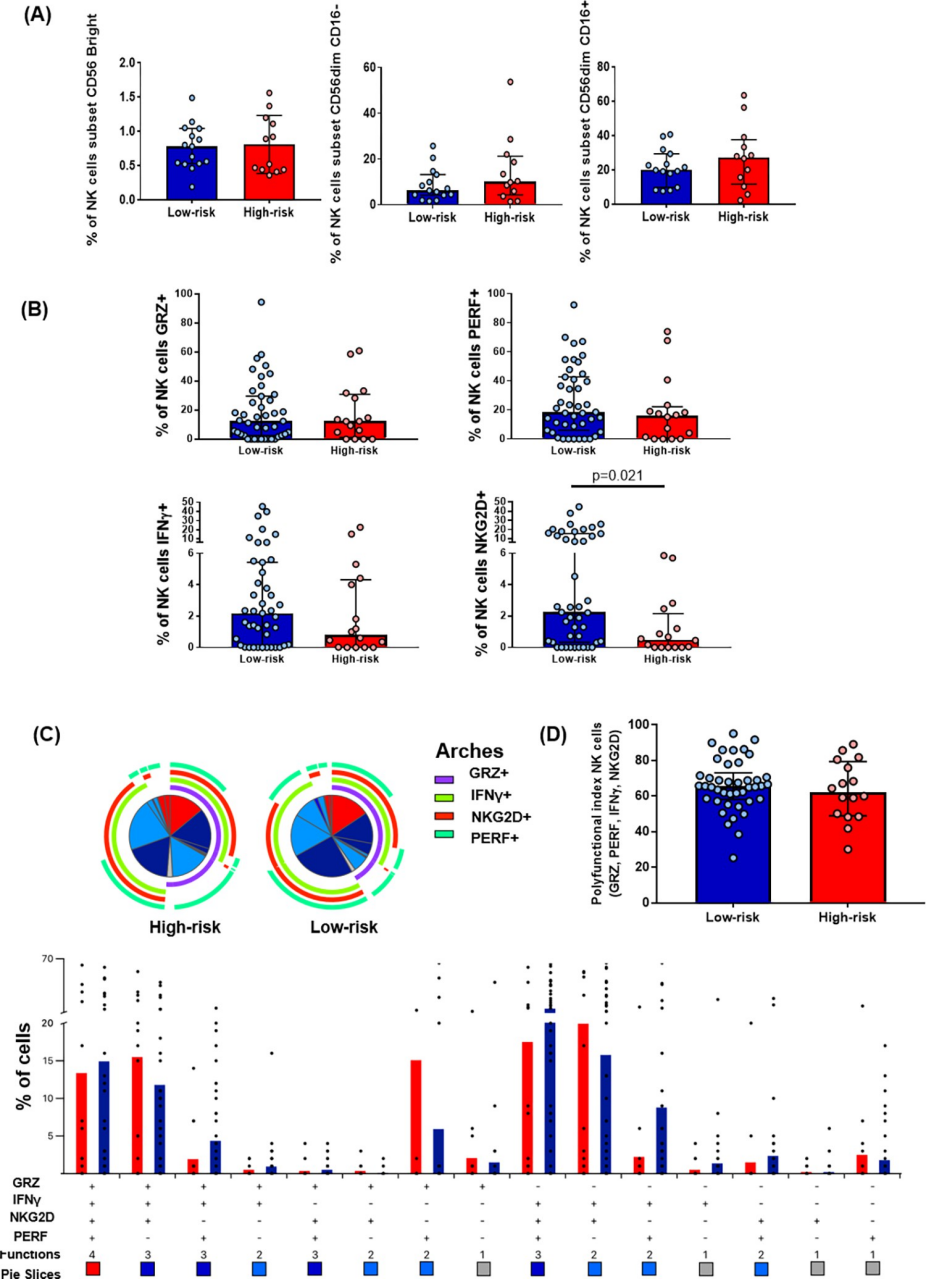
NK cells subset distribution, NKG2D expression, and polyfunctional index. **(A)** Comparison between both MSM groups of NK cells subsets (CD56bright, CD56dim CD16+, CD56dim CD16−) from fresh peripheral blood. The comparison was realized using the Mann–Whitney U-test. Low-risk n = 15, high-risk n = 12. **(B)** Comparison of percentage of total NK cells (CD3−CD56+CD16− and CD3−CD56+CD16+ PBMCs) expressing Granzyme B, Perforin, IFN-γ, or NKG2D between both MSM groups. The comparison was realized using the Mann–Whitney U-test. Data are presented after background subtraction. Low-risk n = 43, high-risk n = 14. **(C)** Comparison of the polyfunctional profiles of total NK cells (CD3−CD56+CD16− and CD3−CD56+CD16+ PBMCs) from both MSM groups. The slices of the pies correspond to the proportions of total NK cells expressing 1 (grey), 2 (light blue), 3 (dark blue), or 4 (red) functions, until n simultaneous parameters (n + 1 dimensions) are calculated using the Boolean gating; the results are presented as mean. The permutation analysis conducted in the SPICE platform only included data higher than 0.1% (after background subtraction). **(D)** INDEX of polyfunctionality (pINDEX) of total NK cells from both MSM groups based on the proportions of cells expressing Granzyme B, Perforin, IFN-γ, and NKG2D; the pINDEX was calculated with Funky Cells software; the comparison was performed using the Mann–Whitney U-test. Low-risk n = 43, high-risk n = 14.

The CD56 and CD16 expressions were analyzed in the CD3− cells from fresh peripheral blood to explore the different NK cell subpopulations in both MSM groups (Fig 3A in S1 Text). Both MSM groups showed a similar NK cell subpopulation distribution. Then, the expression of IFN-γ, Perforin, Granzyme B, and NKG2D was assessed in total NK cells from PBMCs after being stimulated for 48 h with the combination of IL-12 and IL-15 (Fig 2 in S1 Text). The high-risk group showed a lower percentage of total NK cells expressing NKG2D (*p* = 0.021). No differences were found in the magnitude or polyfunctional index in those cells (Fig 3).

### No differences were found in the expression of antiviral genes in anal mucosa between both MSM groups

**Fig 4:**
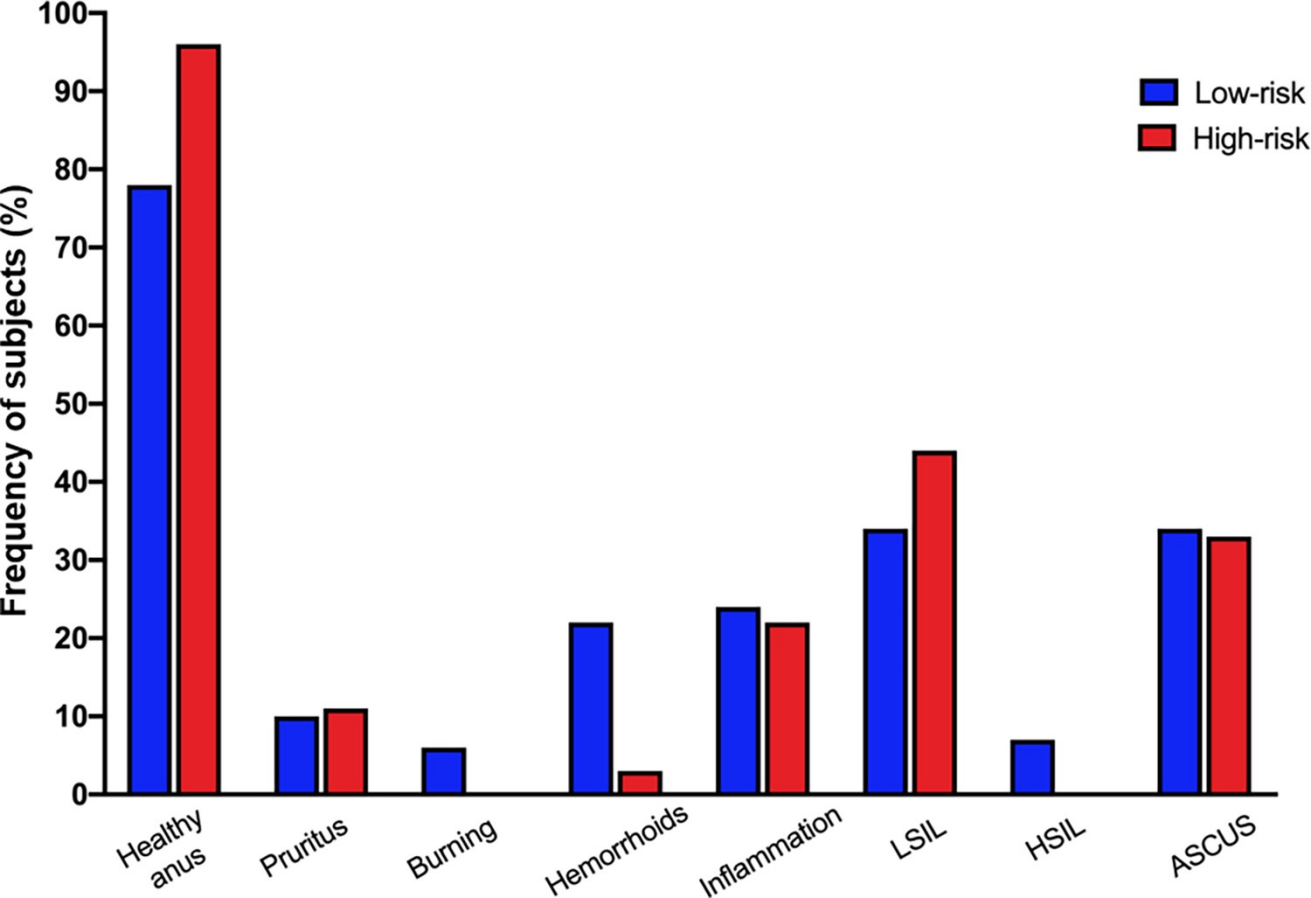
Symptoms, macroscopic, and microscopic findings in anal mucosa tissue of both MSM groups. Comparison of symptoms, macroscopic, and microscopic findings in the anal mucosa tissue of both MSM groups. Data represent the percentages of people presenting each condition. No statistical differences were found between both groups through the Chi-square test. Low-risk (n = 29), high-risk (n = 9). ASCUS: atypical squamous cells of uncertain significance; LISIL: low-grade squamous intraepithelial lesion; HSIL: high-grade squamous intraepithelial lesion.

An anal sampling was made to explore the macroscopic and microscopic state of anal mucosal tissue and the expression of the antiviral genes *HPN1*, *HBD2*, *HBD3*, *SLPI*, *RNAse7*, *TRIM5α*, and *APOBE3G*. When reviewing the state of the anus, it was observed that most of the subjects in both MSM groups presented no symptoms and a healthy anus. In the microscopic analysis of anal cells (cytology) both groups showed a low frequency of intraepithelial lessons. No statistical differences were found regarding symptoms or the macro and microscopic findings of anal mucosa between both groups (Fig 4). A low amount of mRNA was obtained from the anal mucosa samples, so it was only possible to make a comparison of the mRNA detection from the antiviral genes and their expression could not be quantified. No statistical differences were found between the MSM groups regarding the detected antiviral genes HPN1, HBD2, HBD3, SLPI, RNAse7, TRIM5α, and APOBE3G (Table 4 in S1 Text).

## Discussion

This study reached a population with challenging access, given the conditions of the LGBTI population vulnerability in our country [32] and the inclusion criteria that directed the recruitment of individuals with very high-risk sexual behaviors in the absence of sex work. These results and our previous study showed that MSM individuals in the high-risk group are at extreme risk of HIV infection, showing behaviors that exceed by far the factors associated with seroconversion in the other MSM cohorts, who remain seronegative [30]. No correlations were observed between the immunological variables and the sexual behaviors of MSM; however, significant differences were found between both groups regarding immune factors that have been previously associated with resistance to HIV-1 and that limit its transmission/acquisition.

The MSM high-risk group for HIV infection showed a low activation profile of T and NK cells. The lower T cell activation profile has been previously described in HESN, which has low expression of T cell activation markers [33] and higher percentages of Treg cells [34]. The study of Camara *et al*. [35] showed that HESN of serodiscordant couples had a lower percentage of CD4+ T cells expressing CD38 than control subjects. A similar low percentage of CD4+ T cells positive for CD38 has been described in MSM HESN from an Amsterdam cohort [33], and in HESN from female sex workers of the Pumwani cohort in Kenya [23], as well as in Colombian elite controllers [36]. Moreover, the low percentages of T cells expressing activation molecules are related to lower susceptibility to HIV-1 infection *in vitro*, and the persistent HIV-1 seronegative status is associated with lower T cell activation [33, 37].

Our group has previously described that elite controllers exhibit a lower percentage of NK and T cells expressing activation molecules than HIV-1 progressors [38], which points to a protective quiescent cell profile characterized by a low activation of immune cells without the loss of functionality together with the aforementioned studies. These findings are similar to our cohort, in which a low T-cell activation profile with a strong response to SEB superantigen was observed. Surprisingly, some individuals showed a T-cell response to HIV-1 peptides, but not differences in this response were found between the MSM groups. A CTL response is expected in infected individuals, but not in individuals without evidence of established infection, such as HESN. Nevertheless, a CTL response has been associated with natural resistance to HIV-1 in previous studies and has been observed in serodiscordant couples from Spain [39, 40], India [41] and Senegal [42]; and in heterosexual Caucasian, African American and Hispanic HESN-women [43]. No other studies have reported low expression of NKG2D in HESN nor elite controllers; however, Muntasell *et al*. demonstrated that viral exposure in a model of CMV infection resulted in a decreased NKG2D expression, which selectively limited the ability of NK cells to kill target cells that express high levels of NKG2D ligands while preserving the expression of other NK activation molecules and the NK cytotoxic potential [44]. Similarly, we previously found that MSM at high risk from this cohort showed higher cytotoxic capacity and IFN-γ production in response to K562 cell stimuli compared to MSM at low risk of HIV-1 infection [45]. These findings reinforce the theory that the quiescent immune profile is a protective factor associated with the control of HIV-1 and is not limited to T cells. To our knowledge, this is the first evidence of low NKG2D expression by NK cells associated with natural resistance to HIV-1 infection in HESN.

A higher Serpin A1 expression in PBMCs was found in the high-risk group. High levels of Serpin A1 have previously been described in the genital mucosa of HESN female sex workers [46], genital and oral mucosa of HESN serodiscordant couples, and GALT tissue of HIV-1 elite controllers [9]. The anti-HIV function of Serpin A1 in the mucosa is well understood because it prevents tissue integrity damage, thereby avoiding the inflammatory response and transmigration of the virus to other tissues [47, 48]. The anti-HIV effect of Serpin A1 in peripheral blood is significant although less discussed, considering that this serine protease (i) inhibits neutrophil elastase, which promotes the entry of HIV-1 into the cell through its binding to gp120 and through the cleavage that makes SDF-1 (CXCL12) and CXCR4, thereby facilitating the binding of HIV-1 with this receptor; (ii) inhibits gp120 formation; and (iii) inhibits p55 processing to p24 by protease [48]. This anti-HIV effect has been previously demonstrated by the absence of HIV infection in the whole blood compared to the presence of infection in the lymphoid nodules under the same *in vitro* conditions [49], as well as in the easier replication of the virus in the whole blood of individuals with inherited Serpin A1 deficiency, compared to the lower infection rate in the whole blood of healthy controls [50]. To our knowledge, this is the first evidence of increased Serpin A1 expression by PBMCs in seronegative MSM cohorts at high risk of HIV-1 infection.

Our study has limitations, such as the lack of a proper low-risk control group, for example, MSM in exclusively monogamous relationships or who always use condoms. Moreover, the small sample size, no probabilistic sampling, and the cross-sectional nature of the study do not allow us to infer and extrapolate our findings to other populations, as well as build an explicative and causality model of HIV-1 resistance in MSM at high risk of HIV-1 infection.

Collectively, this suggests that Colombian MSM at high-risk could be HESN individuals, and natural resistance against HIV-1 could be a combination of quiescent T and NK cell profiles and increased Serpin A1 expression by PBMCs. Further study of MSM at high risk of exposure to HIV-1 is necessary to better understand their natural response to the virus and improve the prevention and therapeutic strategies against HIV-1 infection.

## Supporting information

S1 Text The "S1 Text" file contains all the supporting tables and figures. (DOCX)

S1 Graphical abstract (TIF)
