# Supplementary material for: Seronegative MSM at high risk of HIV-1 acquisition show an immune quiescent profile with a normal immune response against common antigens
Source: PLoS One. 2022 Dec 8;17(12):e0277120. doi: 10.1371/journal.pone.0277120 (PMC9731495; doi:10.1371/journal.pone.0277120)
Supplement: S1 Text — (DOCX) [file pone.0277120.s001.docx]

**Supplementary Material**

**S1 Text**

**Table 1.** Specific primers and profiles for real-time PCR of PBMC samples

| **Gene** | **Primer** |
| --- | --- |
| PGK | Fw 5` GTTGACCGAATCACCGACC 3`  Rv 5` TCGACTCTCATAACGACCCGC 3` |
| Foxp3 | Fw 5` ACCTTCCCAAATCCCAGTGC 3`  Rv 5`CCTGGCAGTGCTTGAGGAAGT 3` |
| Elafin | Fw 5` AAACACCTTCCTGACACCATGA 3`  Rv 5` TTAACAGGAACTCCCGTGACAG 3` |
| Serpin A1 | Fw 5´ CCGCCATCTTCTTCCTGCCTGA 3´  Rv 5´ CCGGAGAGGTCAGCCCCATTG 3´ |
| IL-1β | Fw: 5´ GGATATGGAGCAACAAGTGG3`  Rv: 5´ ATGTACCAGTTGGGGAACTG3` |
| IL-18 | Fw: 5´ ATGGCTGCTGAACCAGTAGAAG3`  Rv: 5´ CAGCCATACCTCTAGGCTGGC3` |
| Caspase 1 | Fw5´ CAAGGGTGCTGAACAAGG 3`  Rv5´ GGGCATAGCTGGGTTGTC 3` |

**Table 2.** Specific primers and profiles for real-time PCR for anal mucosal samples

| **Gene** | **Primer** |
| --- | --- |
| PGK | Fw 5` GTTGACCGAATCACCGACC 3`  Rv 5` GAGACCACAGGTGCCAATTT 3` |
| HBD-2 | Fw 5` ATCAGCCATGAGGGTCTTGT 3`  Rv 5`CCTGGCAGTGCTTGAGGAAGT 3` |
| HBD-3 | Fw 5` ATCTTCTGTTTGCTTTGCTCTTCCTGTTTT 3`  Rv 5` AGCACTTGCCGATCTGTTCCTCCTT 3` |
| HNP-1 | Fw 5´ GCAAGAGCTGATGAGGTTGC 3´  Rv 5´ GTTCCATAGCGACGTTCTCC 3´ |
| Cathelicidin | Fw` 5` GGATGCTAACCTCTACCGC 3`  Rv`5` AGGGTCACTGTCCCCATACA 3` |
| SLP-1 | Fw` 5` GATGTTGTCCTGACACTTGTGG 3`  Rv` 5` GCAT TTC CAG CCA GAC AGA T 3` |
| Trim5-α | Fw` 5` TTCTGTCAGGAGGACGGGAA 3`  Rv` 5` GCTTCTGCCTCAGCATCTC 3` |
| APOBEG-3 | Fw` 5` TCTTTGTTGCCCGCCTCTAC 3`  Rv` 5` CACGAACTTGCTCCAACAGTG 3` |
| RNAse -7 | Fw` 5` AACAGACACAGCGTAGCCC 3`  Rv` 5` GGCAGGGGTCGCTTTGC 3` |

**Fig 1. Gating strategy for CD4+ and CD8+ T cells in the activation profile assay**


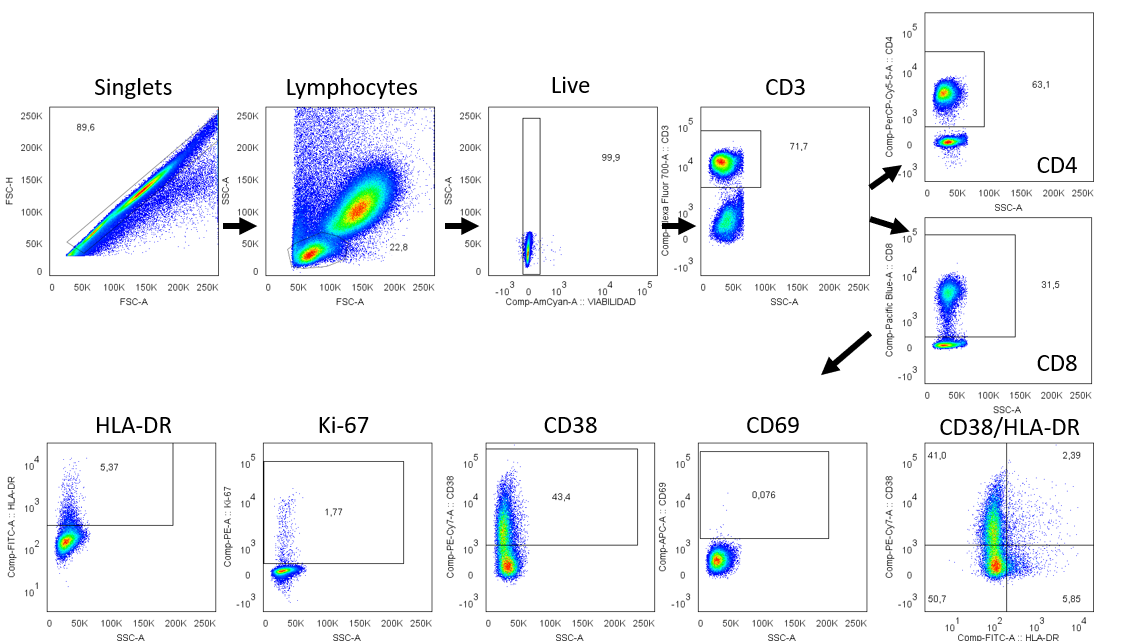
T cells from fresh peripheral blood. At least 100.000 events were read at lymphocytes gate. The number inside each gate correspond to percentage of cells.

**Fig 2. Gating strategy for CD4+ and CD8+ T cells in the HIV-1-specific T cells responses assay**

T cells from PBMCs cultured with HIV-1 Gag peptides or SEB. At least 100.000 events were read at lymphocytes gate. The number inside each gate correspond to the percentage of cells.

**
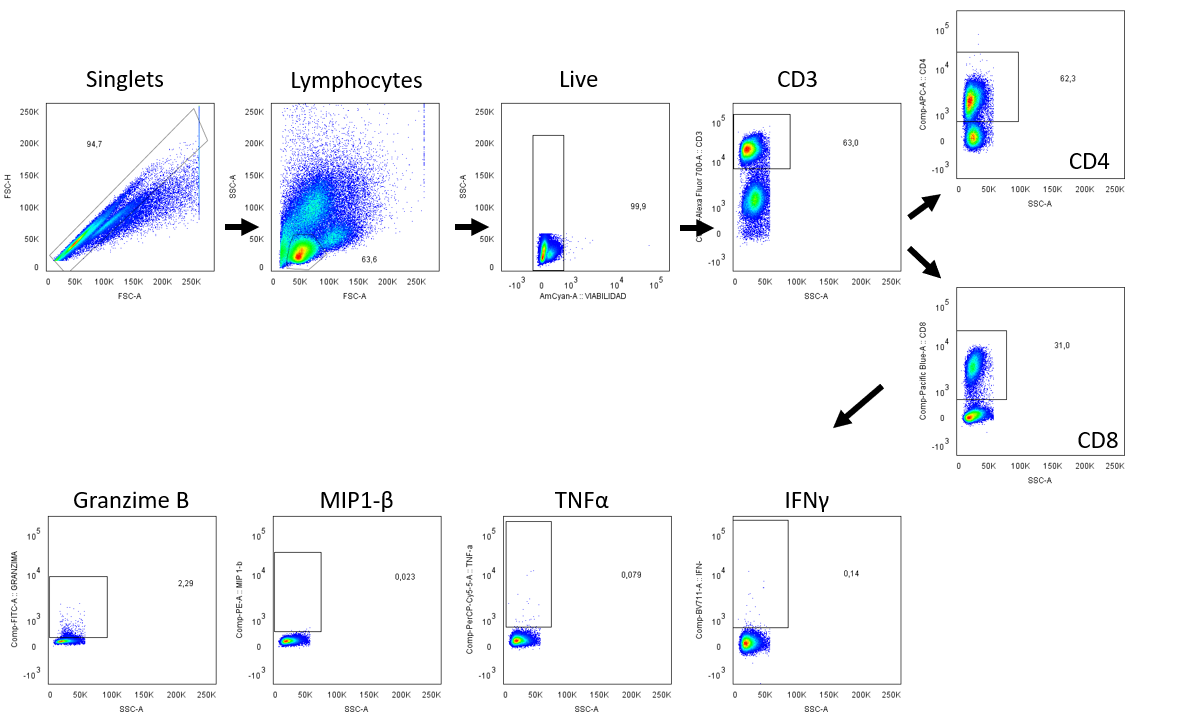
**

**Fig 3. Gating strategy for NK cells**

(A) NK subpopulations from fresh peripheral blood. (B) NK gating from PBMCs cultured with IL-12 and IL-15. At least 100.000 events were read at lymphocytes gate. The number inside each gate correspond to the percentage of cells.

(A)


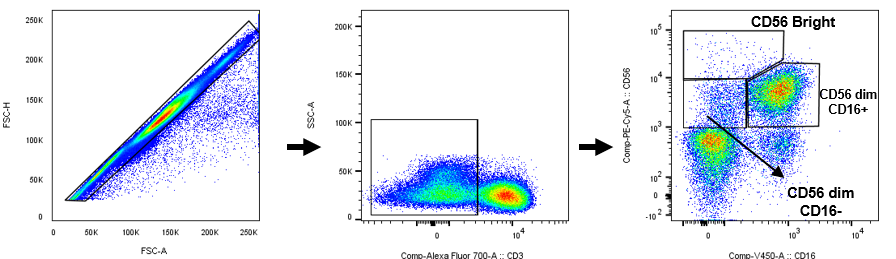


**
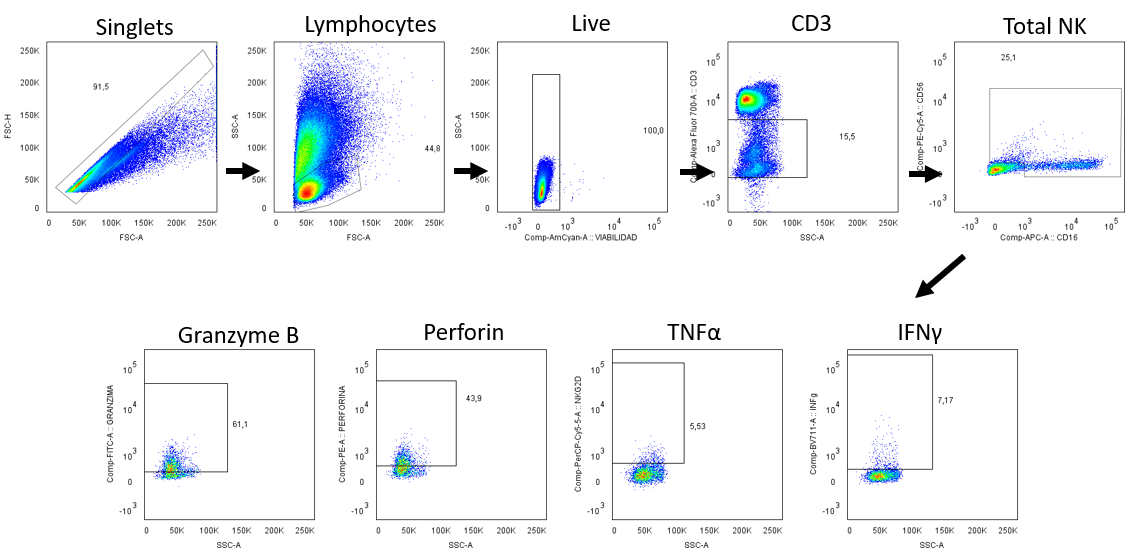
**(B)

**Table 3. Levels of cytokines in supernatants of HIV-1 stimulated cells from both groups of MSM.**

| **Cytokine** | **Low-risk**  **(n=44)** | **High-risk**  **(n=16)** | **U Mann Whitney** |
| --- | --- | --- | --- |
|  | **pg/mL Median (IQR)** | **pg/mL Median (IQR)** | **p-value** |
| **IL-1β** | 64,765 (35,872-97,948) | 52,916 (14,487-77,179) | 0.31 |
| **IL-6** | 8,845 (6,932-9,082) | 9,082 (8,519-9,082) | 0.17 |
| **IL-8** | 22,249 (17,653-24,745) | 24,147 (19,389-27,717) | 0.32 |
| **IL-10** | 32 (31.9-32.1) | 32.1 (32-57.95) | 0.06 |
| **IL-12** | 3.9 (3.6-4.4) | 4.6 (3.8-22.55) | 0.10 |
| **TNF**-**α** | 462.1 (93.65-1,008) | 894.7 (439.9-10,540) | 0.30 |

**Table 4. mRNA detection of antiviral genes in anal mucosal tissue of both groups of MSM**

| **Gene** | **Low-risk** | **High-risk** | **Fisher** |
| --- | --- | --- | --- |
|  | **n=29** | **n=9** | **p value** |
| **HPN1** | 9 | 2 | 1,00 |
| **HBD2** | 8 | 2 | 1,00 |
| **HBD3** | 7 | 1 | 0,65 |
| **CATELICIDIN** | 3 | 0 | 1,00 |
| **SLPI** | 12 | 2 | 0,44 |
| **RNASE 7** | 7 | 1 | 0,65 |
| **TRIM5 ALFA** | 3 | 2 | 0,57 |
| **APOBE3G** | 5 | 2 | 1,00 |
