# Supplementary figures and images for: Seronegative MSM at high risk of HIV-1 acquisition show an immune quiescent profile with a normal immune response against common antigens
Source: PLoS One. 2022 Dec 8;17(12):e0277120. doi: 10.1371/journal.pone.0277120 (PMC9731495; doi:10.1371/journal.pone.0277120)

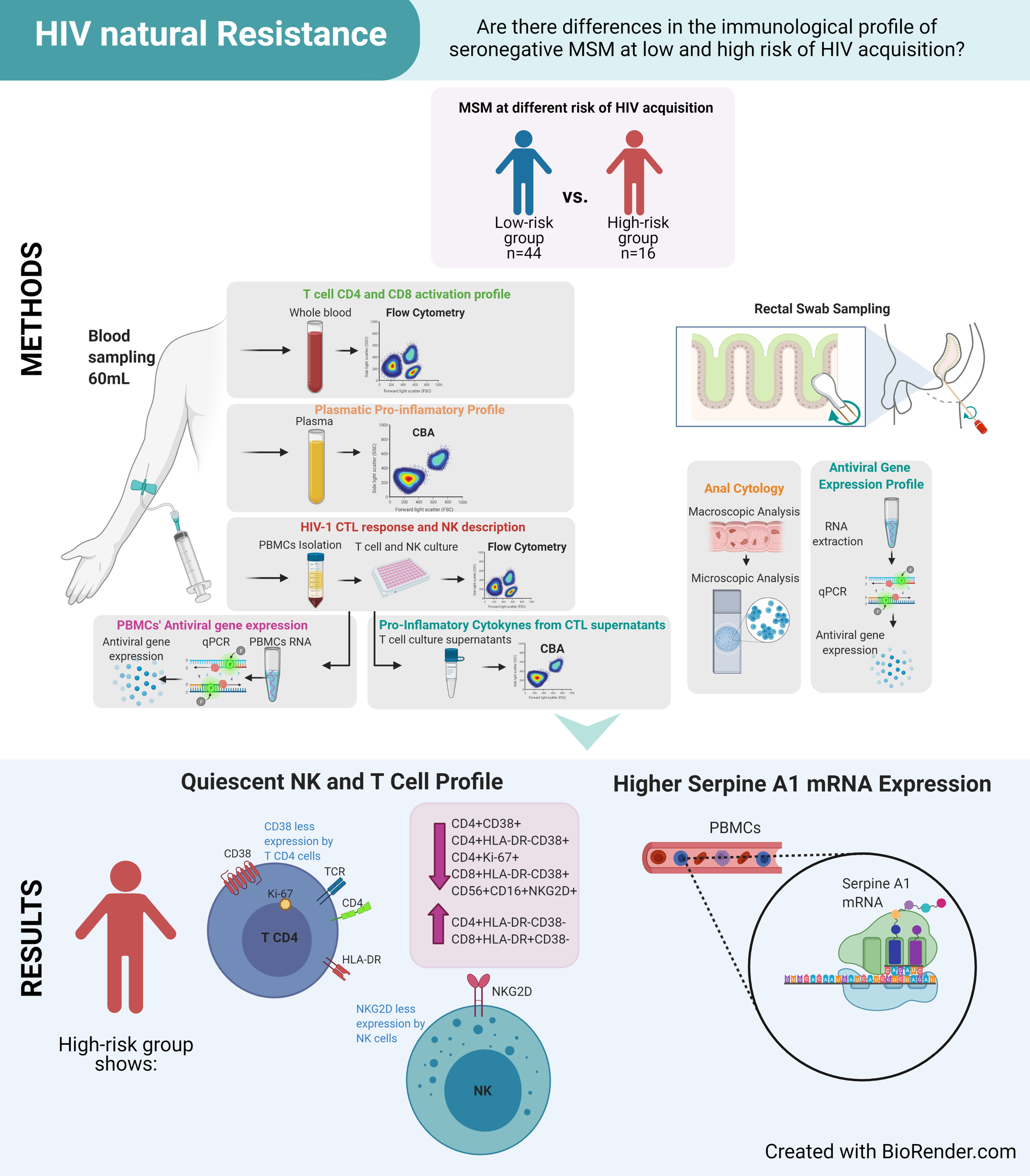

Supplement: S1 Graphical abstract — (TIF) [file pone.0277120.s002.tif]
